# Chylous Ascites: A Rare Initial Presentation of High‐Grade Follicular Lymphoma

**DOI:** 10.1155/crom/5559043

**Published:** 2026-05-03

**Authors:** Rama Nada, Ani Gvajaia, Ali Raza, Mohammed Raji

**Affiliations:** ^1^ Department of Internal Medicine, New York City Health and Hospitals Corporation (NYCHHC), Lincoln Medical and Mental Health Center, Bronx, New York, USA, nychealthandhospitals.org; ^2^ Division of Hematology and Oncology, Henry Ford Health System, St John Hospital, Detroit, Michigan, USA, henryford.com; ^3^ Department of Surgical Oncology, New York City Health and Hospitals Corporation (NYCHHC), Lincoln Medical and Mental Health Center, Bronx, New York, USA, nychealthandhospitals.org; ^4^ Department of Pathology, New York City Health and Hospitals Corporation (NYCHHC), Lincoln Medical and Mental Health Center, Bronx, New York, USA, nychealthandhospitals.org

**Keywords:** chylous ascites, follicular lymphoma, lymphadenopathy, lymphoma, non-Hodgkin, pleural effusion, rituximab

## Abstract

**Background:**

Chylous ascites is an uncommon condition characterized by the accumulation of triglyceride‐rich, milky fluid in the peritoneal cavity due to lymphatic disruption or obstruction. Although lymphoma is a leading cause of malignant chylous ascites, its occurrence as an initial presentation remains rare.

**Case Presentation:**

A 69‐year‐old woman presented with a three‐month history of postprandial abdominal pain, weight loss, anorexia, and dyspnea. Imaging revealed extensive abdominal and pelvic lymphadenopathy with bilateral pleural effusions. Diagnostic laparoscopy demonstrated milky peritoneal fluid, and fluid analysis confirmed chylous ascites (triglycerides, 1361 mg/dL). Lymph node biopsy demonstrated high‐grade B‐cell lymphoma with morphological features favoring follicular lymphoma. Immunohistochemistry revealed a markedly elevated Ki‐67 proliferative index (> 90%), and genomic profiling identified pathogenic *EZH2* and *TET2* mutations with a high tumor mutational burden. According to the fifth edition of the WHO Classification of Haematolymphoid Tumours (2022), these findings are most consistent with follicular lymphoma, a mature B‐cell neoplasm with high‐grade features. Given the high‐output drainage and recent surgery, cytotoxic chemotherapy was deferred, and rituximab monotherapy was initiated. Rapid clinical improvement and decreased drain output allowed safe transition to standard R‐CHOP therapy, achieving a complete metabolic response (Deauville score 2) after six cycles.

**Conclusions:**

This case highlights chylous ascites as a rare but important presenting feature of lymphoma. Its recognition should prompt early histopathologic evaluation and multidisciplinary management. In selected postoperative or frail patients, rituximab monotherapy can serve as an effective bridge to full chemotherapy, facilitating recovery and improving outcomes. Early diagnosis and targeted treatment remain essential to prevent complications from lymphatic loss and to optimize prognosis in lymphoma‐associated chylous ascites.

## 1. Introduction

Chylous ascites is an uncommon clinical entity characterized by the accumulation of lipid‐rich, milky‐appearing fluid in the peritoneal cavity, typically resulting from lymphatic disruption or obstruction, most often involving the thoracic duct or abdominal lymphatics [1]. This condition is frequently associated with malignancy, infection, or trauma. Although older series estimate the overall incidence of chylous ascites at approximately 1 in 20,000 hospital admissions, more recent data are lacking [2]. In cases of malignant chylous ascites, lymphoma, especially non‐Hodgkin lymphoma, is among the most common etiologies, often accounting for approximately one‐half of cases [3]. Less commonly, gastrointestinal, gynecologic, or other solid tumors may contribute through similar mechanisms.

Chylous ascites typically suggests extensive lymphatic involvement and often correlates with advanced‐stage disease. Its presence may delay or complicate chemotherapy initiation, particularly in patients with poor performance status or significant protein loss [4].

In this report, we describe the case of a 69‐year‐old woman who initially presented with postprandial abdominal pain, significant weight loss, and intraoperative discovery of chylous ascites. Histopathology confirmed high‐grade B‐cell lymphoma with morphological features favoring follicular lymphoma (FL). The patient achieved a favorable clinical response after rituximab followed by R‐CHOP therapy. This case underscores the importance of early recognition and multidisciplinary management of chylous ascites in suspected lymphoma.

## 2. Case Report

A 69‐year‐old woman presented with a three‐month history of postprandial abdominal pain, described as intense, localized to the mid‐abdomen, and radiating to the back. She reported associated unintentional weight loss of 25 pounds, anorexia, malaise, dyspnea, and recent melena. She denied nausea, vomiting, fever, or diarrhea. Her medical history was significant for diabetes and prior cholecystectomy. On physical examination, she had abdominal distension and periumbilical tenderness. Laboratory evaluation, including hepatic function tests and lipase, was unremarkable.

**Figure 1:**
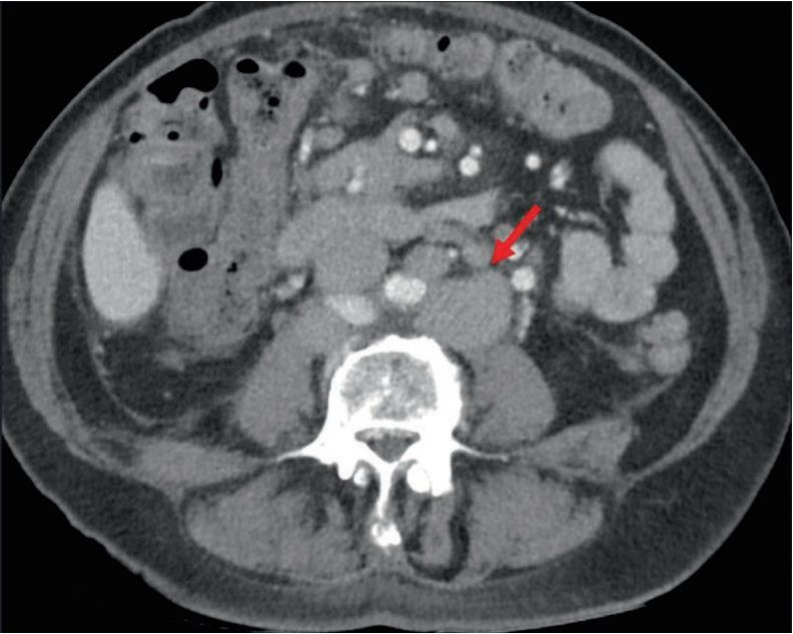
Lymphadenopathy in the para‐aortic lymph nodes seen on contrast‐enhanced CT; biopsy confirmed follicular lymphoma.

**Figure 2:**
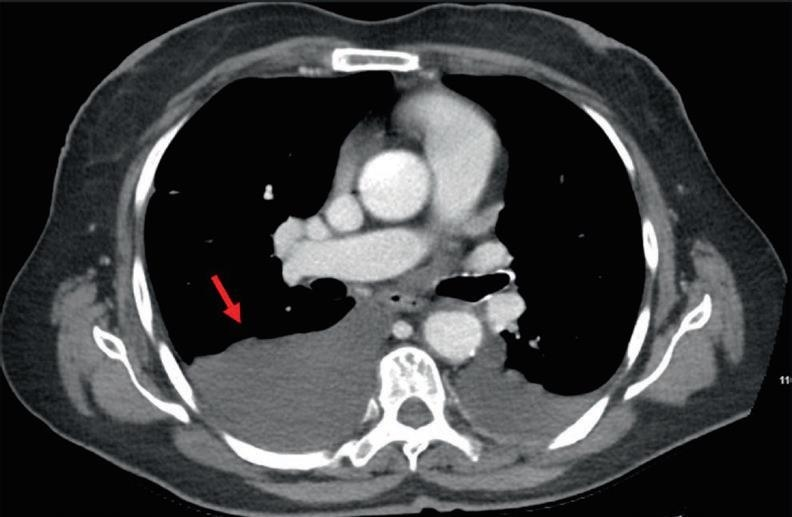
Bilateral pleural effusions on contrast‐enhanced CT of the chest, found incidentally and later confirmed to be chylous.

CT imaging of the chest, abdomen, and pelvis demonstrated moderate‐volume ascites with extensive lymphadenopathy involving the gastrohepatic ligament, periportal, para‐aortic, retrocaval, and mesenteric regions, with representative nodes measuring up to approximately 1.5–2.0 cm in short axis (Figure 1). Additional findings included bilateral perirectal and iliac chain lymphadenopathy and bilateral pleural effusions (Figure 2). Thoracentesis confirmed the presence of chylous pleural effusion.

**Figure 3:**
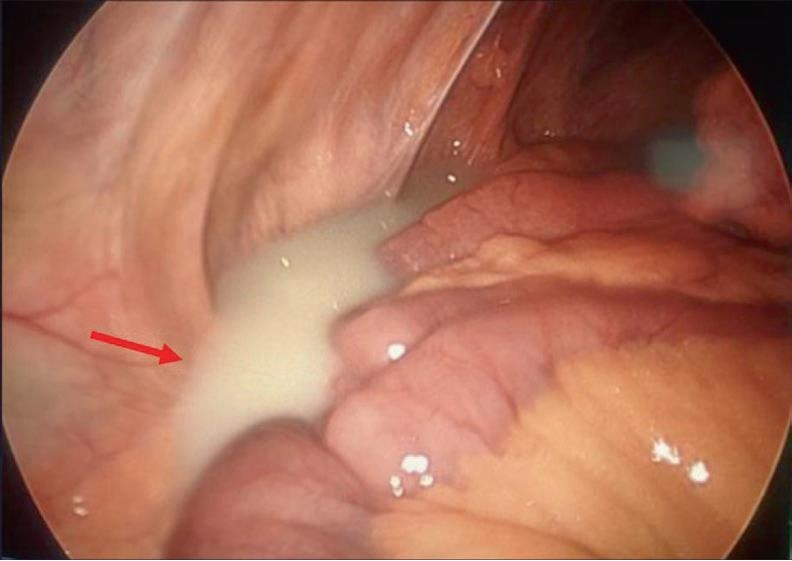
Intra‐abdominal chylous ascites identified during diagnostic laparoscopy.

Because of worsening abdominal pain during hospitalization, the patient underwent robotic‐assisted laparoscopic exploration with adhesiolysis. Biopsies were obtained from enlarged perigastric, colonic mesenteric, and left iliac lymph nodes. Intraoperatively, milky peritoneal fluid suggestive of purulence was noted (Figure 3), along with a phlegmonous reaction near the duodenum. Concern for a sealed duodenal perforation led to conversion to an open laparotomy, which confirmed a sealed duodenal ulcer. Three Jackson–Pratt (JP) drains were placed.

**Figure 4:**
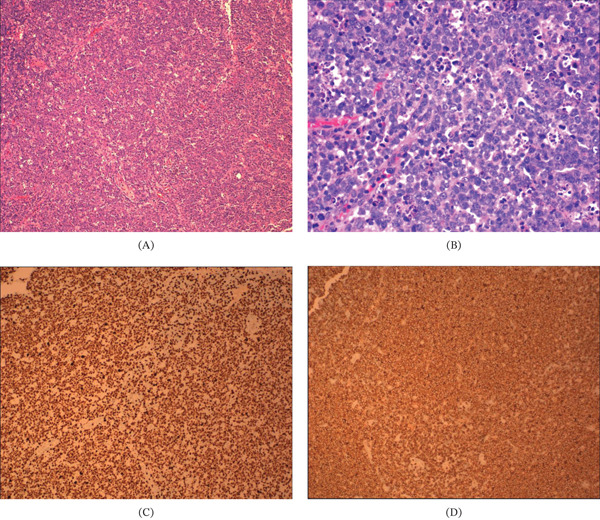
Histopathologic findings of lymph node biopsy. (A) Hematoxylin and eosin–stained section demonstrating a high‐grade malignant lymphoma, favoring follicular lymphoma with unusual features (original magnification ×100). (B) Higher‐power H&E view showing brisk mitotic activity and prominent apoptotic bodies (original magnification ×400). (C) Ki‐67 immunohistochemical stain demonstrating a markedly elevated proliferative index, with staining in > 95% of tumor cells. (D) CD20 immunohistochemical stain showing strong membranous positivity in tumor cells, consistent with B‐cell lineage.

Postoperative fluid analysis revealed a triglyceride level of 1361 mg/dL, confirming chylous ascites. Biopsy of a colonic mesenteric lymph node demonstrated high‐grade B‐cell lymphoma with unusual cytological features, morphologically favoring FL. Immunohistochemical analysis revealed a markedly elevated Ki‐67 proliferative index (> 90%) in tumor cells (Figure 4A–D). Genomic testing identified a pathogenic EZH2 p.(Tyr646Phe) mutation, a TET2 p.(Cys1378Tyr) variant, and a high tumor mutational burden (13.3 mutations/Mb). According to the fifth edition of the WHO Classification of Haematolymphoid Tumours (2022), these findings are most consistent with FL, a mature B‐cell neoplasm with high‐grade features.

Baseline FDG PET/CT demonstrated widespread hypermetabolic lymphadenopathy involving supraclavicular, mediastinal, mesenteric, gastrohepatic, periportal, retroperitoneal, iliac, perirectal, and inguinal nodal stations. The most intense uptake was observed in a left iliac lymph node with an SUVmax of 17.3 and in a peri‐aortic lymph node with an SUVmax of 16.3. Liver uptake measured SUVmean 2.0 (SUVmax 2.4) and was used as a reference.

Although the patient’s initial presentation was characterized by abdominal pain and weight loss, chylous ascites was identified intraoperatively and served as the pivotal finding that ultimately led to the diagnosis of lymphoma.

The disease was staged as Ann Arbor stage IIIB, with no bone marrow involvement. Baseline laboratory evaluation demonstrated mildly elevated lactate dehydrogenase (LDH) at 259 U/L and an elevated *β*2‐microglobulin level of 4.5 mg/L, consistent with increased tumor burden and high‐risk disease features.

Given the high volume of chylous output (approximately 500 mL per drain daily), immediate initiation of cytotoxic chemotherapy was deferred due to concerns regarding impaired wound healing. Conservative management was initiated, including total parenteral nutrition (TPN) and octreotide to reduce lymphatic flow. Despite these measures, chylous output remained persistently elevated. Given the recent laparotomy and ongoing high‐output drainage, rituximab monotherapy was initiated as a bridging strategy to allow clinical stabilization prior to definitive chemoimmunotherapy. Within 5 days, the patient’s symptoms improved and drain output decreased substantially, allowing removal of one drain. By Day 10, another drain had ceased output entirely, and the remaining drain produced only 200 mL/day. The patient was subsequently started on R‐CHOP therapy because of the aggressive disease features. After six cycles, PET/CT demonstrated a Deauville score of 2, indicating a complete metabolic response.

## 3. Discussion

Chylous ascites is a rare but clinically significant manifestation of lymphatic disruption, typically resulting from trauma, infection, or malignancy. The gastrointestinal lymphatic system drains into the thoracic duct through intra‐abdominal and retroperitoneal nodes before entering the systemic circulation. Obstruction or injury at any point along this pathway can lead to chyle leakage into the peritoneal cavity [5, 6].

The thoracic duct, originating from the cisterna chyli and draining into the left subclavian vein, is the principal channel for lymphatic return. Obstruction at various levels can correlate with specific malignancies: retroperitoneal and para‐aortic lymphadenopathy is commonly seen in gastrointestinal cancers; mesenteric lymphadenopathy in pancreatic, gastric, and colorectal cancers; and pelvic lymphadenopathy in gynecologic malignancies. In lymphoma, any nodal group or even the thoracic duct itself may be involved [5].

Although rare, chylous ascites may represent the pivotal clinical clue leading to the diagnosis of an underlying malignancy. Patients frequently present with progressive abdominal distension, weight loss, and discomfort. Diagnosis is confirmed by ascitic fluid triglyceride levels exceeding 200 mg/dL, as seen in our case, in which the level was 1361 mg/dL [7]. Imaging studies often reveal lymphadenopathy and fluid accumulation, but in this patient, chylous ascites was discovered only intraoperatively.

Lymphangiography, including intranodal techniques, and lymphoscintigraphy with SPECT/CT, when available, help localize lymphatic leaks and identify candidates for embolization or surgery [8, 9]. FL is the most common indolent subtype of non‐Hodgkin lymphoma in Western populations [10]. However, its association with chylous ascites may indicate advanced disease and has been linked to poorer prognosis and higher relapse rates after treatment [11].

Pathological confirmation is essential for accurate diagnosis and therapeutic planning [12]. In this case, lymph node histology and immunohistochemistry supported a diagnosis of high‐grade B‐cell lymphoma with unusual cytology but morphology favoring FL [13], with a pathogenic EZH2 Y646 mutation, an established FL driver present in approximately 20%–25% of cases [14]. The very high Ki‐67 index (> 90%) is consistent with aggressive biology [15].

Encouragingly, resolution of ascites after initiation of therapy has been associated with improved outcomes. In a review of 26 cases of lymphoma‐associated chylous ascites, including 10 cases with FL, 11 patients experienced resolution with chemotherapy. Of those, 82% survived, compared with 33% of those with persistent ascites [16].

The prognosis and management of chylous ascites depend on the underlying etiology. Initial treatment involves dietary modification with a high‐protein, low‐fat diet enriched with medium‐chain triglycerides (MCTs), which are absorbed directly into the portal venous system, bypassing the intestinal lymphatics [17]. Response to diet alone is variable, with some series reporting resolution in up to 50% of cases [18]. When conservative measures fail, particularly in refractory or postoperative settings, pharmacologic therapy with somatostatin analogs such as octreotide can reduce lymphatic flow and has shown clinical benefit [19].

In cases of lymphoma‐associated chylous ascites, chemotherapy, radiotherapy, and targeted agents such as rituximab are mainstays of treatment [20]. In our patient, rituximab monotherapy was selected as an initial bridging strategy because of the immediate postoperative setting and persistent high‐output chylous drainage. Initiation of full‐intensity cytotoxic chemotherapy shortly after laparotomy was considered high risk because of concerns regarding impaired wound healing, infection, and ongoing losses of protein and lymphocytes associated with chyle leakage. Although dose‐attenuated chemoimmunotherapy could be considered, even reduced‐intensity regimens carry risks of early myelosuppression and postoperative complications. Given the well‐established sensitivity of FL to anti‐CD20 therapy, rituximab was used as a lower‐toxicity temporizing approach to achieve early cytoreduction while allowing clinical stabilization and nutritional recovery before initiation of definitive R‐CHOP chemotherapy [12, 20].

The response to rituximab in lymphoma‐associated chylous ascites is variable and likely depends on factors such as disease burden, anatomical involvement, and lymphoma subtype. While some cases demonstrate improvement within weeks, others may require longer periods for resolution [16]. In contrast, our patient experienced a rapid reduction in chylous output within 5 days of therapy initiation, allowing earlier transition to definitive chemoimmunotherapy.

Chylous ascites is rich in nutrients and immunoglobulins, and their loss can result in dehydration, malnutrition, electrolyte imbalances, and immunosuppression [1, 17]. Patients with lymphoma‐associated chylous ascites are at higher risk for hospitalization, invasive procedures, and the need for TPN [4, 17]. Early recognition and multidisciplinary management, including oncologic treatment, nutritional support, and surgical input, are critical to improving patient outcomes [6, 17].

This case adds to the limited literature describing chylous ascites as an initial manifestation of high‐grade FL and highlights the potential utility of rituximab monotherapy as a safe and effective bridging approach in postoperative or high‐risk patients. Further investigation into the mechanisms underlying the rapid resolution of lymphatic leakage with B‐cell‐directed therapy may help refine treatment strategies and support the development of standardized management algorithms for lymphoma‐associated chylous effusions.

## 4. Conclusion

Chylous ascites, though uncommon as an initial manifestation, should raise suspicion for underlying lymphoma when accompanied by systemic symptoms and lymphadenopathy. Early pathological confirmation is essential, as timely initiation of targeted therapy can reverse ascites and improve outcomes. In this case, rituximab provided a safe and effective bridge in the immediate postoperative period, enabling subsequent R‐CHOP chemotherapy and achieving complete remission.

## Funding

No funding was received for this manuscript.

## Conflicts of Interest

The authors declare no conflicts of interest.

## Data Availability

Data sharing not applicable to this article as no datasets were generated or analyzed during the current study.
